# Investigating tobacco presence at retail points of sale around schools in Egypt

**DOI:** 10.1186/s12889-025-24675-z

**Published:** 2025-09-24

**Authors:** Eman Hany Elsebaie, Raouf Alebshehy, Amira Haridy Abdelaal, Wael Safwat Abdelmeguid, Doaa Ahmed Saleh

**Affiliations:** 1 Public Health and Community Medicine Department, Faculty of Medicine, Cairo University, Cairo, Egypt; 2 Department for Health, University of Bath, Bath, UK; 3 School of Public Health, Bielefeld University, Bielefeld, Germany; 4 Agriculture Research Center, Animal Health Research Institute, Giza, Egypt; 5 Egypt Health foundation, Cairo, Egypt

**Keywords:** Retail facilities, Schools, Tobacco products, Advertisement

## Abstract

**Objectives:**

This study aims to identify the locations where tobacco products are sold around schools as well as the different marketing strategies followed at the different points of sale (POS).tify

**Methods:**

This study was conducted in five Egyptian governorates (Cairo, Giza, Qualiubia, Sharkia and BeniSuif) representing different geographical regions in Egypt. An observational study was conducted around 102 schools from July to September 2024. The study examined location and products marketing tactics in retailers within 100 m of selected schools.

**Results:**

The study revealed that over half of retailers surrounding the surveyed schools sold at least one type of tobacco and nicotine product. Nearly all schools studied had at least one tobacco POS within a 100-meter radius. All surveyed tobacco POS displayed tobacco products. Different tactics of marketing to children and youth were identified.

**Conclusions:**

School-age children in Egypt face exposure to tobacco products and advertising. Policymakers must act urgently by enforcing advertising bans and restricting product availability, especially around schools, to protect youth from harm.

**Supplementary Information:**

The online version contains supplementary material available at 10.1186/s12889-025-24675-z.

## Introduction

In many markets, the tobacco industry focus on the point of sale (POS) as a critical channel to advertise and promote their tobacco products, which might have been the industry tactic to undermine and bypass the progress in policies prohibiting tobacco advertising via traditional media such as radio, television and on billboards [1]. In the USA, by the end of the 1990s, a number of restrictions on tobacco advertising were adopted but did not include a ban on advertising at POS; within 10 years of the advertising restriction, POS advertising accounted for over three-fourths of tobacco marketing expenditure in the country [2]. In many countries, tobacco advertising and promotion at POS extends beyond product display to include indoor and outdoor advertisements, branded walls and shelves and other branded store accessories [3].

Tobacco companies have historically targeted children and youth with tobacco product advertising. In an internal document from 1990, R.J. Reynolds staff were encouraged to set up promotions at stores frequented by young people, noting that “those stores can be in close proximity to colleges or high schools” [4]. Extensive tobacco advertising at POS near schools has been documented in different setting and there is evidence that stores where youth shop regularly display more advertising than others [5]. Tobacco advertising at POS is associated with youth smoking initiation and progression to regular use [2]. Children and youth who have frequent exposure to tobacco advertising and promotion at POS have higher odds of already having tried smoking and of being susceptible to smoking than those not frequently exposed [6]. POS advertising also discourages cessation efforts and normalizes smoking [7].

Egypt is one of the only six countries worldwide with an increasing tobacco market with currently a quarter of its adults using tobacco and half of its population potentially exposed to secondhand smoke [8]. Smoking rates are significantly high among males and are expected to rise from 40.6% in 2022 to 45% by 2050 [9]. It’s a big country in the Eastern Mediterranean Region (EMR) with the proportion of people under 18 years of about 37.3% [10]. It represents a big market for tobacco presence, attracting the largest tobacco companies to promote their deadly products to youth and catch a new generation of addicts [11]. Even though Egypt became a Party to the World Health Organization Framework Convention on Tobacco Control (WHO FCTC) and prohibits the sale of tobacco products to persons under the age of 18, no policies exist to prohibit sales or promotion within a considerable distance from schools, or any youth gatherings [12]. There is an apparent inconsistency between the prevalence of smoking in Egypt and the tobacco control policies launched in recent years, which emphasizes the need for exploring youth access to tobacco products [13]. Despite our knowledge of tobacco advertising targeted to children and youth near schools, our understanding of the extent of tobacco advertising and promotion across Egypt is deficient. This study aims to investigate the tactics targeting youth in Egypt by identifying the locations where tobacco products are sold around schools as well as the different marketing strategies followed at the different POS.

## Methods

### Type and setting

An observational descriptive study was conducted in five Egyptian governorates (Cairo, Giza, Qualiubia, Sharkia and BeniSuif) representing different geographical regions in Egypt from July to September 2024. The study methodology was adopted from similar studies done within Johns Hopkins University and Campaign for Tobacco Free Kids Tiny Target Project [14, 15]. Specifically, we employed a standardized observation checklist and implemented a systematic walking strategy, covering a 100-meter radius surrounding each school to assess tobacco POS.

### Sample

The study examined location and products marketing tactics in retailers within 100 m of different types of schools; A multistage sample technique was used. First, a convenient sample of 5 Egyptian governorates was *purposively selected to capture diverse geographical*,* economic*,* and cultural contexts through representing different regions in Egypt; Cairo*,* Giza*,* Qualiubia representing urban governorates; Sharkia representing Lower Egypt; and BeniSuif representing Upper Egypt ensuring a broad representation despite the non-probabilistic sampling approach*. Secondly, a random sample from the districts in each governorate was selected. A total of 55 districts were included, approximately 10 per governorate, except for Cairo, which had 15. Within each district, around two schools were selected based on accessibility. Schools were chosen to reflect variability in proximity to tobacco retailers (within 100–250 m). Finally, *two schools were selected from each district based on convenience (accessibility)*, we tried to include all grades (primary, preparatory, and secondary) and types (governmental, private, and international). Proportional representation was not feasible due to logistical constraints (e.g., data availability, permissions), but we ensured coverage of key strata (e.g., urban/rural, school size). A total sample of 102 schools was selected.

### Data collection, analysis, and synthesis

Ten data collectors were recruited and trained to collect data on the practices of marketing and promotion of tobacco, nicotine and alternative products at POS around schools. A team of two data collectors went to each selected school, where they explored the area around the school by following a systematic walking pattern starting from the front entrance of the selected schools; walked through and observed all streets within the 100 m-radius as shown on the geographic mapping application; identified different retail stores while distinguishing between retailers that sell or do not sell tobacco; described the type of retailers (shopping malls, kiosks, handheld carts, …). Retail stores that sell tobacco is referred to as POS in this study.

An observation checklist was created to collect data from the different observed POS. Kobo Toolbox was used as a tool for online data entry based on the developed checklist. An online geographic mapping application was used to identify the exact location of the selected school. Maps were created for each 100-meter sampling area surrounding the schools using a free mapping and distance measurement tool available at https://www.acscdg.com/. This tool’s distance calculator was used to estimate measurements and generate consistent reference maps. These maps were then used to implement the walking protocol and identify/observe tobacco POS within the sampling areas.

At each identified tobacco POS, data collectors used an online observation form to document various details. Collected data was uploaded to a cloud-based database in real time. Data included the types of products sold, such as cigarettes, molasses and/or waterpipe, electronic cigarettes (e-cigarettes), and heated tobacco products (HTP), as well as the forms in which the products were sold (e.g., single cigarettes, unpackaged mollasses). The presence of any type of product advertisement or promotion was also noted, with particular attention to displays of cigarettes near snacks, sweets, and sugary drinks, and advertisements placed at children’s eye level (approximately one meter off the ground). Additionally, the display of flavored cigarettes was documented. A monitoring and quality check of the data collected was conducted in real time, before leaving the school area, to verify and complete any missing information.

The data were coded and cleaned on a data sheet prepared in Excel. The Statistical Package for Social Sciences (SPSS version 27) was used for data analysis to ensure accurate and comprehensive results. All collected data were revised for competencies and logical consistency. Simple descriptive statistics of mean, median and standard deviation was used for the summary of numerical variables, while frequencies and percentages were used for categorical ones. The data were tabulated and presented primarily in the form of graphs for clear visualization of the findings. Descriptive statistics were used to summarize key findings.

## Results

### Retailers and products

**Fig. 1:**
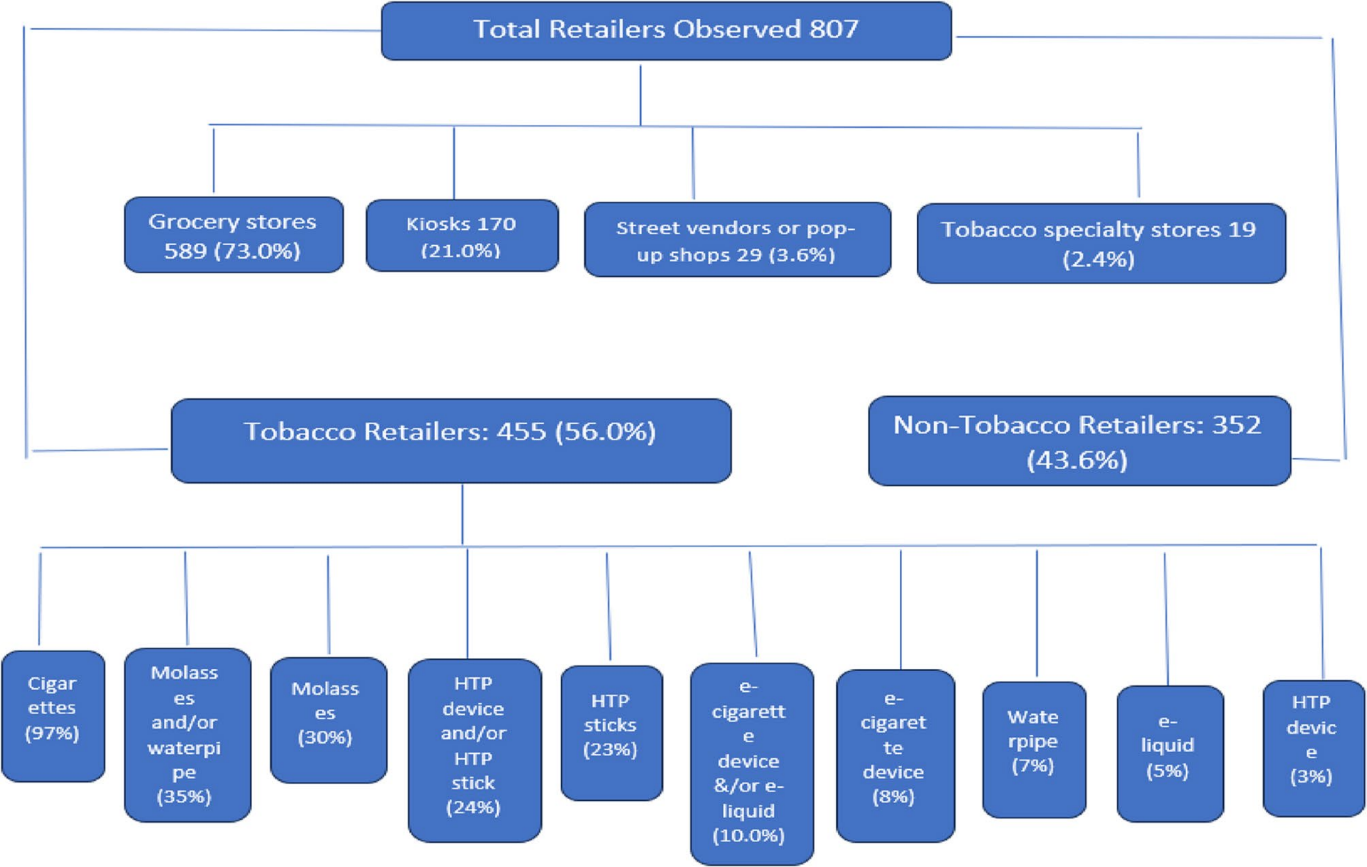
Access to Tobacco Products Around Schools: Retailer Types and Density

A total sample of 102 schools was surveyed in urban and rural areas in five Egyptian governorates. Two schools were located far from residential areas and had no surrounding POS. One school had three retail stores within a 100-meter radius, none of which sold tobacco products. The remaining 99 schools had at least one POS selling a type of tobacco products. The total observed retail stores were 807 distributed into 589 grocery stores (supermarkets or mini markets), 170 kiosks, 29 street vendors or pop-up shops, and 19 tobacco specialty stores. Of the 807 retailers observed, a total of 455 retailers (56.0%) sold at least one type of tobacco and nicotine products, classifying them as tobacco POS. There were between one and sixteen tobacco POS per school and half of the schools had more than four tobacco POS (median = 4) within 100-meter radius, with each school having an average of about five tobacco POS (mean = 4.8, SD = 3.3). It was found that 56.2% of the POS selling cigarettes also sold flavored cigarettes, and 11% of those selling HTP sticks sold flavored HTP sticks (Fig. 1)

### Products placement and display

All types of tobacco and nicotine products sold in the studied POS (*n* = 455) were recorded. The most common products available for purchase were cigarettes (97.0%) followed by molasses and/or waterpipe (35.0%), molasses (30.0%), HTP device and/or HTP stick (24.0%), HTP sticks (23.0%), e-cigarette device &/or e-liquid (10.0%), e-cigarette device (8.0%), waterpipe (7.0%), e-liquid (5.0%) and HTP device (3.0%) (Table 1).

**Table 1:** Types of tobacco products sold in the observed retailers within 100 m radius of schools

Tobacco products	*N* (%) *N* = 455
Cigarettes	441 (97.0%)
Molasses &/or Waterpipe	159 (35.0%)
Molasses	138 (30.0%)
HTP device &/or HTP stick	108 (24.0%)
HTP sticks	106 (23.0%)
E-cigarette device &/or Eliquid	44 (10.0%)
E-cigarette device	35 (8.0%)
Waterpipe	33 (7.0%)
E-liquid	23 (5.0%)
HTP device	14 (3.0%)

It was observed that various tobacco packets were arranged on their sides, and therefore it was impossible to see the health warnings and pictorial health warnings that were on the front and back of each pack. As a result, warnings were only visible on the packs in 37.1% of POS that displayed cigarettes and 40.0% of those that exhibited molasses. On the other hand, just 3% of POS that sold HTP sticks had health warnings visible on them.

**Fig. 2:**
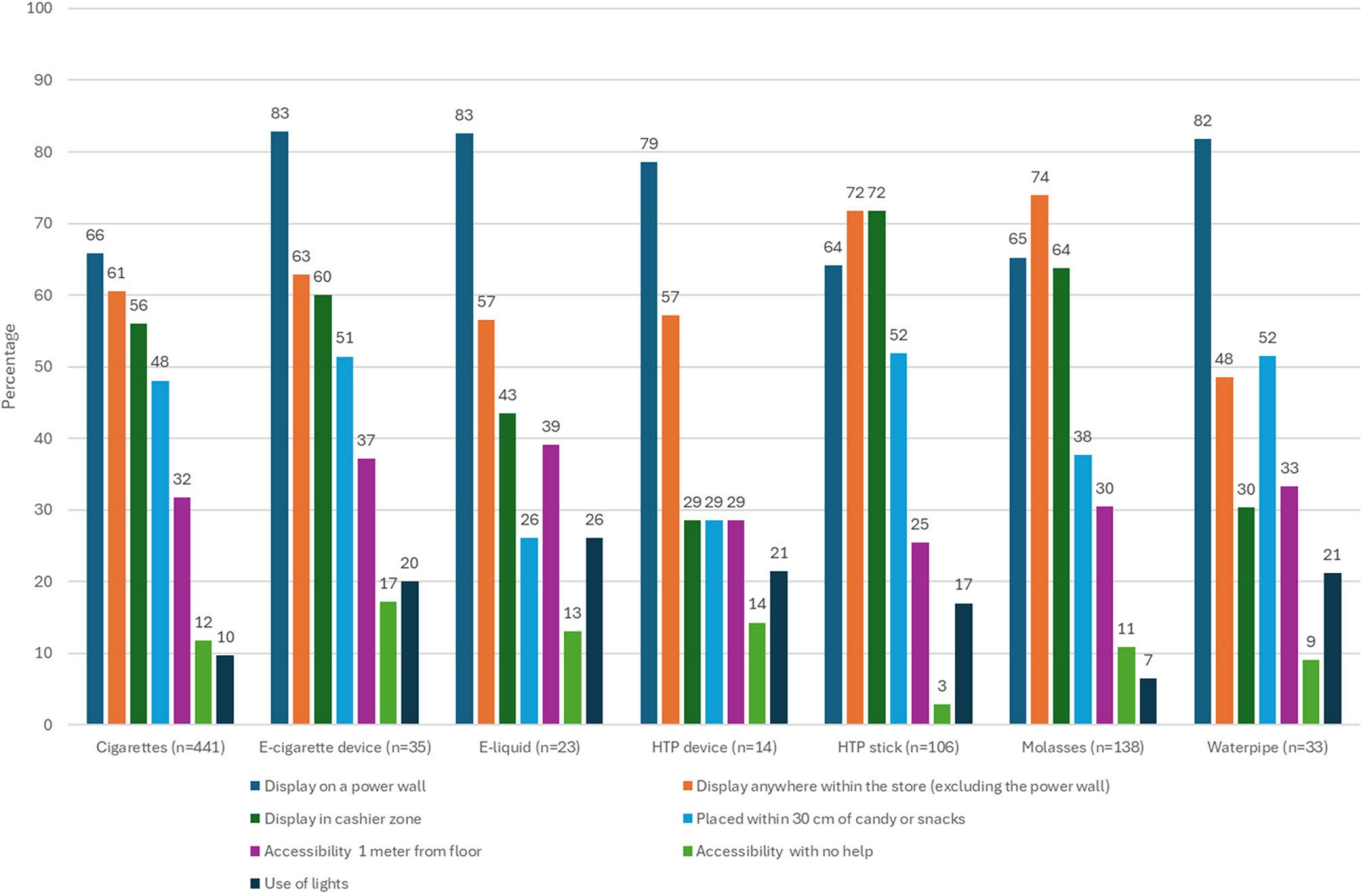
Forms of products’ display recorded in the observed POS within a 100-meter radius of schools

Products available for purchase were on display in all tobacco POS. Various forms of product displays were documented at the observed POS, including on power walls in 67.0%, in other areas within the store 61.5% and in cashier zones 56.4%. Displaying products within 30 centimeters of candy and snacks were observed in 48.5% of POS, while 46.5% displayed the products at the eye level of children. Products were accessible without the help of the vendor in 12.7% of POS, while 11.4% used lights to show the products. Forms of different products’ display recorded in the observed POS are presented in Fig. 2.

### Practice regarding age and single sticks restrictions

Only one POS displayed a sign indicating that selling tobacco to minors was prohibited; and it did not specify the age limit. Of the observed POS selling cigarettes, 85.0% sold single cigarette sticks, while 20.0% of those selling molasses sold unpackaged molasses.

### Marketing, advertising, and promotion of products

Of the observed POS, 35.0% featured one or more types of advertisement either inside or outside the store. The most common form of advertising inside the POS was print signage apart from display, with 17.9% of cigarette advertisements falling into this category. E-cigarettes and HTPs had lower percentages; 5.7% and 14.3%, respectively. Electronically illuminated advertisements were present in 9.4% of HTP stick promotions. Digital signage was notably used for HTP sticks (7.5%). Many advertisements were positioned at a low height, with 10.4% of cigarette advertisements placed within one meter of the floor, enhancing visibility for potential customers. Outside the POS, advertisements on the façade were noted, particularly for cigarettes (11.8%) and molasses (12.3%). Cigarettes had the highest overall advertising presence, followed by HTP sticks and molasses. E-cigarettes and other products showed lower promotional activity. Only one POS was found to offer free cigarette samples. Table 2 summarizes forms of advertising for each product observed at POS.

**Table 2:** Forms of advertising for each product observed at POS within a 100-meter radius of schools

	Cigarettes (*n* = 441)	E-cigarette device (*n* = 35)		E-liquid (*n* = 23)	HTP device (*n* = 14)	HTP stick (*n* = 106)	Molasses (*n* = 138)	Waterpipe (*n* = 33)
Advertisement inside the POS								
Print signage (apart from display)	79 (17.9%)		2 (5.7%)	1 (4.3%)	2 (14.3%)	19 (17.9%)	3 (2.2%)	2 (6.1%)
Print signage (part of display)	14 (3.2%)		-	-	1 (7.1%)	17 (16.0%)	-	-
Electronically illuminated ads	10 (2.3%)		-	-	-	10 (9.4%)	-	-
Digital signage	7 (1.6%)		1 (2.9%)	1 (4.3%)	1 (7.1%)	8 (7.5%)	-	-
3D signage	11 (2.5%)		-	-	-	4 (3.8%)	-	-
Ads for products use cartoon characters	1 (0.2%)		-	-	1 (7.1%)	1 (0.9%)	-	-
Ads observed within 1 m from floor	46 (10.4%)		2 (5.7%)	2 (8.7%)	2 (14.3%)	18 (17.0%)	3 (2.2%)	1 (3.0%)
Advertisement outside the POS								
Advertisements on the façade of the POS	52 (11.8%)		-	1 (4.3%)	-	13 (12.3%)	7 (5.1%)	-
Print signage outside the store	38 (8.6%)		-	-	-	10 (9.4%)	-	-
Illuminated/backlit ads outside the store	9 (2.0%)		-	-	-	7 (6.6%)	-	-
3D ads outside the store	7 (1.6%)		-	-	-	-	-	-
Display of ads of products that are inside the store visible from outside the store (through a window, door, or storefront)	29 (6.6%)		-	1 (4.3%)	-	15 (14.2%)	-	1 (3.0%)

## Discussion

Tobacco use in Egypt remains a critical public health challenge, with high prevalence rates persisting despite tobacco control efforts. A key driver of this issue is the tobacco industry’s documented strategy of targeting youth to sustain and expand its market. Notably, Egypt is among the six countries globally where the tobacco market continues to grow. In this context, our study aimed to identify and expose the tobacco and nicotine marketing practices employed at POS near schools.

Our findings revealed that over half (56%) of retailers surrounding the surveyed schools sold at least one type of tobacco and nicotine product. Moreover, nearly all schools studied had at least one tobacco POS within a 100-meter radius. These results are consistent with global research documenting the concentration of tobacco POS near schools, typically within 100–250 m, in countries such as Argentina, Bangladesh, Bulgaria, Georgia, Kyrgyzstan, and Romania [16]. This pattern reflects the tobacco industry’s deliberate targeting of future generations, particularly in low- and middle-income countries (LMICs) [14].

In terms of the types of tobacco and nicotine products sold at the studied POS, a wide range of products was recorded. These findings align with global data showing that the tobacco industry continues to prioritize its traditional products, such as cigarettes, while simultaneously introducing newer products like HTPs and e-cigarettes. This dual strategy reflects the industry’s effort to maintain its traditional markets while expanding its reach by promoting alternative products, thereby ensuring market growth and diversification [17].

Regarding the marketing of tobacco and nicotine products at the observed POS around schools, all surveyed POS displayed tobacco products prominently, and approximately one-third featured advertisements or special promotions. This finding is consistent with the literature [14], which highlights the prevalence of promotional items for tobacco products, particularly cigarettes, in retail environments. Studies have shown that despite restrictions on tobacco advertising in many contexts, the retail environment remains a key channel for tobacco marketing, especially in areas frequented by youth [14, 18, 19]. Evidence suggests that efforts to reduce youth exposure to tobacco marketing have led to the adoption of restrictions on advertising in some domains [20]; however, these restrictions often exclude retail settings. As a result, advertisements and promotions for tobacco and nicotine products persist in stores, contributing to youth exposure and the normalization of addiction [21]. Egypt, a Party to the WHO FCTC, has made progress in tobacco control by adopting advertising bans. However, indirect advertising remains pervasive, particularly through depictions of tobacco use in films and television dramas [22]. This indicates insufficient enforcement of existing regulations, allowing the tobacco industry to exploit gaps in policy implementation and maintain its marketing presence, especially in environments accessible to youth [12].

Regarding product placement and display, various forms of displays were observed, including prominent placements on power walls and in cashier zones. Notably, almost half of the POS displayed tobacco products within 30 centimetres of candy and snacks (48.5%) and placed them at the eye level of children (46.5%). These findings align with global evidence, which has documented that in 90% of the countries examined, cigarettes were displayed in prominent locations, such as in front of or on the counter, making them easily accessible to both adults and minors [14]. Furthermore, the literature shows that tobacco advertisements and product displays at the eye level of children, approximately one meter off the ground, are a common tactic employed at POS [6]. This strategy aims to increase visibility and appeal to younger consumers, highlighting the deliberate targeting of children. These findings underscore the urgent need for stricter regulations on product placement and display at POS, particularly in areas frequented by children, to reduce their exposure and prevent early initiation into tobacco use [14].

Regarding health warnings and pictorial health warnings on displayed products, our study found that these warnings were visible on the packs in only around 37% of POS displaying cigarettes and 40% of those displaying molasses. This is consistent with the literature, which highlights significant challenges in the implementation and enforcement of pictorial health warning policies, even after the adoption of relevant laws. These challenges often include issues with quality of printing, image manipulation, tax stamp placement, packaging design and text manipulation [23]. Our study identified the additional challenge of product placement withing display settings to hide the warnings. The limited visibility of warnings at POS undermines the intended impact of these policies. We however suggest avoiding any form of products display.

Regarding flavored products, our study found that approximately 56.2% of the POS selling cigarettes also sold flavored cigarettes, and 11% of the POS selling HTP sticks offered flavored HTP sticks. These findings align with evidence from a study conducted across 42 countries, which observed the presence of flavored cigarettes and/or flavored cigarette advertisements at POS near schools in 76% of the countries examined [14]. The widespread availability of flavored tobacco products near schools is particularly concerning, as flavors are well-documented to appeal to youth by masking the harshness of tobacco and enhancing its attractiveness [24]. This creates an added risk of early initiation and sustained use. To address this issue, stronger enforcement of regulations is necessary to eliminate the availability and marketing of flavored tobacco products, especially in proximity to schools.

Referring to age restriction signage, Egypt’s tobacco control law mandates that individuals in charge of stores selling tobacco are also obligated to post clear signs in prominent places at the selling points to indicate ban of tobacco selling to under 18-year-olds [12]. Despite this requirement, our study found that only one POS displayed a sign indicating that selling tobacco to minors was prohibited; however, it did not specify the age limit. Additionally, the study revealed that approximately 85% of the POS selling cigarettes also sold single cigarette sticks, while around 20% of those selling molasses offered unpackaged molasses. These findings align with the literature, which indicates that the sale of single cigarette sticks is a common practice in most LMICs [14]. This practice not only undermines tobacco control measures but also increases accessibility and affordability for minors, facilitating early initiation and sustained tobacco use.

These findings highlighted a critical gap between policy and practice in Egypt’s tobacco control efforts. While Egypt has established legal frameworks to restrict youth access to tobacco, implementation remains inadequate. The absence of proper signage suggests that vendors either disregard the law or are unaware of their obligations. Additionally, the widespread sale of single sticks and unpackaged molasses points to a loophole that needs addressing, as these practices make tobacco products more accessible to minors.

The WHO Global Report on Trends in Prevalence of Tobacco Use (2000–2030) estimates that at least 37 million adolescents aged 13–15 years are current users of tobacco products, with approximately 10% of adolescents in this age group globally reporting the use of one or more forms of tobacco [8]. This is a concerning indication of a growing tobacco market among youth, which aligns with Egypt’s status as one of the few countries experiencing an expanding tobacco market. These findings underscore the urgent need for stricter tobacco control policies and enforcement, particularly in regulating the proximity of tobacco POS to schools.

Policymakers must take immediate and stronger action to protect children and adolescents from the harmful influence of tobacco marketing. The recommended retail reduction measures in the literature [25], supported by Egypt’s public opinion [26], including banning tobacco sales near schools, reducing numbers of tobacco retailers, prohibiting product displays at POS, and strengthening enforcement of existing laws, are essential to reducing youth access to tobacco.

This study is subject to limitations that may affect the generalizability and accuracy of its findings. The use of a non-probability convenience sampling method might have the possibility of selection bias, as the selected schools may not represent the broader population across Egypt. Additionally, the study’s limited geographic scope, focusing only on five governorates, may not fully capture regional variations in tobacco and nicotine marketing practices. The three-month data collection period may also overlook seasonal trends or special events that influence products sales and marketing, for example during or outside school terms. As this is an observation study, observer bias is another concern, as data collectors may have different interpretations or inconsistencies during observations, despite training. The study’s focus on a 100-meter radius around schools may miss tobacco and nicotine marketing outside this range, which could still affect student exposure. These limitations should be considered when interpreting the results of the study, as there may be additional marketing tactics targeting school-age children that were not captured in this research.

### Strengths and limitations

- The study employs a multistage sampling approach and systematic geographic mapping, ensuring accurate data collection on tobacco marketing near schools.
- The real-time data-collection allows for immediate data entry and quality control, enhancing reliability and reducing data loss.
- Given Egypt’s growing tobacco market and lack of restrictions on sales near schools, the study provides critical insights into youth exposure to tobacco marketing, can inform stronger tobacco control measures.
- The purposive selection of five governorates (Cairo, Giza, Qaliubia, Sharkia, and Beni Suef) ensured regional diversity; however, the findings may not be generalizable to all of Egypt. Within the selected areas, the use of puposive sampling poses a risk of excluding marginalized groups, and the absence of probabilistic methods limits the study’s statistical generalizability.
- This study focused on a descriptive analysis of tobacco marketing practices. Future research could build on these findings by quantitatively examining the relationship between specific POS strategies (e.g., product placement, promotions, proximity to schools) and sales data, to more directly assess their impact.

## Conclusion

**Fig. 3:**
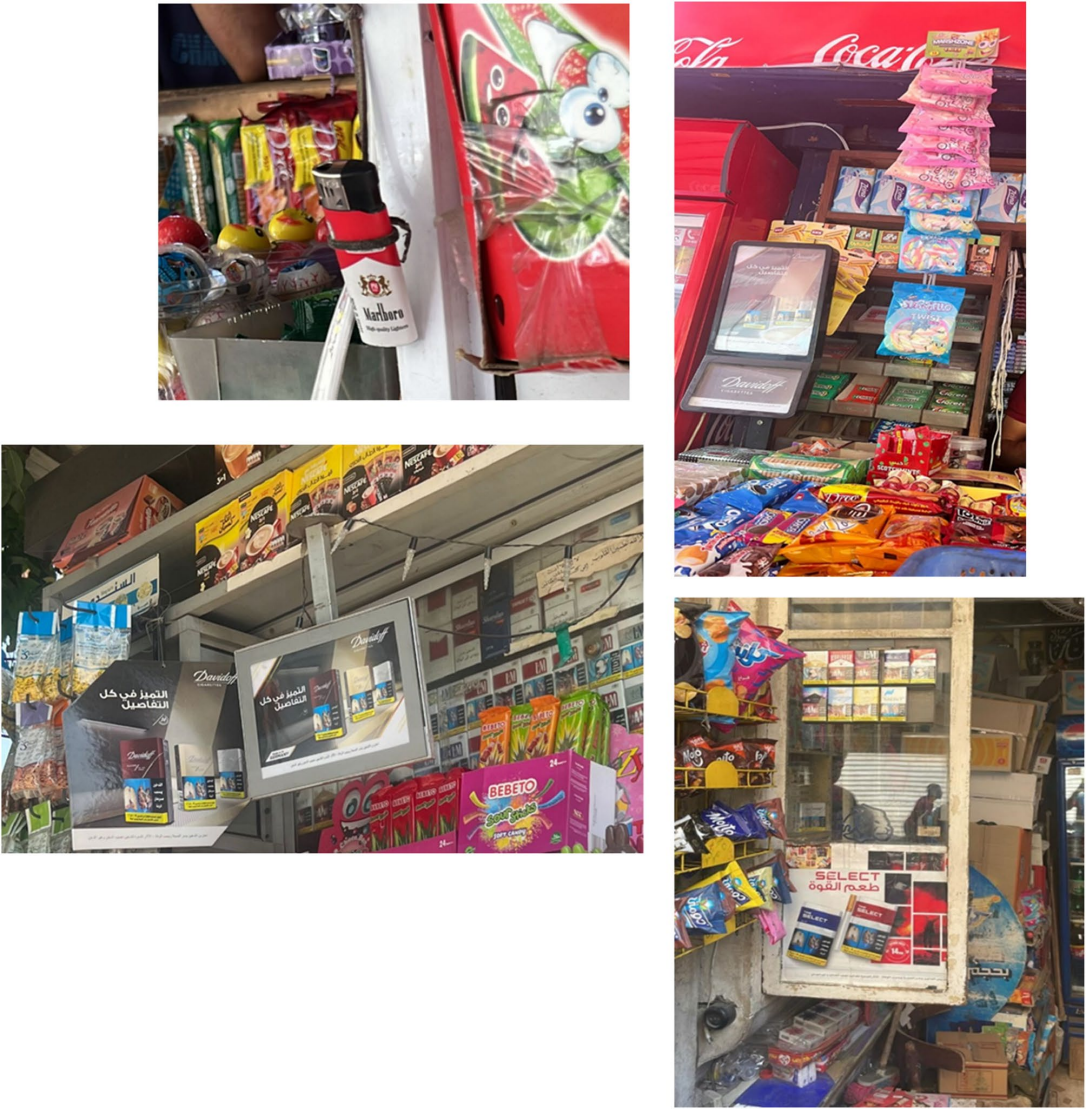
Visual Evidence of Tobacco Retail Practices Around Schools in Egypt

This study reveals alarming trends in tobacco accessibility and marketing near schools in Egypt. Many retailers engaged in practices that increase affordability and appeal to minors (e.g., single-stick sales, lax age verification), directly undermining tobacco control efforts. Despite progress in national policies, the pervasive exposure of youth to tobacco near schools remains a critical public health challenge (Fig. 3).

## Supplementary Information

Supplementary Material 1.

Supplementary Material 2.

## Data Availability

No datasets were generated or analysed during the current study.
