# Supplementary material for: Investigating tobacco presence at retail points of sale around schools in Egypt
Source: BMC Public Health. 2025 Sep 24;25:3055. doi: 10.1186/s12889-025-24675-z (PMC12459026; doi:10.1186/s12889-025-24675-z)
Supplement: Supplementary file 1 — Supplementary Material 1. [file 12889_2025_24675_MOESM1_ESM.pdf]

**BEFORE ENTERING THE STORE:**
1. Observer name/ ID
2. Date
3. Governorate
4. City
5a. School level
5b. School type
6. School name
7. Observed Outlet ID
8. Enter street and number of the venue
9. Retailer Type
9(a). Are you allowed to enter the facility?
9(b). Can you see inside from outside and able to make observations on product display and advertisements?
**PRODUCT PLACEMENT/DISPLAY**
10. What types of tobacco and/or nicotine products do the retailer sell?
11. Are types of tobacco and/or nicotine products displayed on a power wall?
12. Are both the health warning labels and pictorial health warnings on the cigarettes visible?
13. Are the health warning labels and pictorial health warnings on the pack of Molasses visible?
14. Are the health warning labels on the E-liquid visible?
15. Are the health warning labels on the pack of sticks visible?
16. Are there any product displays (anywhere within the store) for cigarettes/ e-cigarettes/ HTPs/ WPT?
17. Are cigarettes/ e-cigarettes/ HTPs/ WPTP displayed in the cashier zone?
18. Are cigarettes/ e-cigarettes/ HTPs/ WPT accessible to the consumer without help from a cashier or store attendant?
19. Are cigarettes/ e-cigarettes/ HTPs/ WPT that are accessible to the consumer without help placed within 1 meter from the floor?
20. Is more than one cigarette pack displayed per brand?
21. Are lights used to illuminate cigarettes/ e-cigarettes/ HTPs/ WPT?
22. Are cigarettes/ e-cigarettes/ HTPs/ WPT placed within 30 cm (estimate using your leg or body) of candy, beverages or snacks?
**PRODUCT PROMOTIONS & ADVERTISEMENTS**
23. How are the cigarettes/ e-cigarettes/ HTPs/ WPT advertised?
• Print signage (posters, banners, flyers, etc.), apart from a product display
• Print signage inside/as part of a product display (backgrounds, shelf liners)
• Digital signage (video or electronic screen)
• Electronically illuminated advertisements
• Three-dimensional signage
• None of the above
• Unknown
24. Are any advertisements for cigarettes/ e-cigarettes/ HTPs/ WPT placed within 1 meter from the floor?
25. Do any advertisements for cigarettes/ e-cigarettes/ HTPs/WPT use cartoon characters?

26. Do advertisements for cigarettes/ e-cigarettes/ HTPs/ WPT include any of the following messages?
• Can be used for smoking cessation
• Less Harmful than cigarettes
• None of the above
• Unknown
27. Are cigarettes/ e-cigarettes/ HTPs/WPT promoted in any of the following ways?
• Price discounts (e.g. sales, coupons, limited time offer, multipack discounts)
• Free samples (with or without the purchase of other goods)
• Cigarette pack/lighter exchanges
• Gifts other than the product (free or with purchase)
• A tobacco brand sponsored event, activity, or cause
• Contests or competitions
• Loyalty/rewards scheme
• Presence of brand representative
• Direction to social media channel
• QR code to website
• None of the above
• Unknown
28. Do you see any cigarettes/ e-cigarettes/ HTPs/WPT branding on non-tobacco products in the store (e.g., clocks, hats, t-shirts, mirrors, carry bags, cigarette lighters, counter mats)?
**FLAVORS**
29. Does the store sell any flavored cigarettes?
30. Flavored e-cigarettes?
31. Flavored HTPs?
**OTHER SIGNAGE**
32. Is there a sign that says cigarettes can only be sold to adults?
**SINGLE STICK SALES**
33. Does the retailer sell single sticks of cigarettes?
34. Does the retailer sell unpackaged Molasses?
**OUTSIDE THE STORE: PRODUCT ADVERTISEMENTS**
35. Are there any advertisements for cigarettes/ e-cigarettes/ HTPs/WTP on the façade of the retailer?
36. How are the cigarettes/ e-cigarettes/ HTPs/WTP advertised?
• Print signage (posters, banners, flyers, etc.), apart from a product display
• Backlit or illuminated advertisements
• Three-dimensional signage
37. Are any of the following items related to cigarettes/ e-cigarettes/ HTPs/WPT that are inside the store visible from outside the store (through a window, door, or storefront) ?
• Display of products (including power wall)
• Advertising
• None of the above
• Not applicable

38. Are there any cafes or restaurants near this school that serve Shisha?
39. Are there any advertisements for cigarettes/ e-cigarettes/ HTPs/WPT on the façade of the café/restaurant?
**COMPLETION OF OBSERVATION**
40. If you can take a picture without drawing unwanted attention, please take a picture of the outside of the store.
41. Do you have any additional notes on your observations in this store?
42. Would you recommend this vendor for professional photography?
43. Result of observation
44. If observation is not completed, why?
45. What are the other reasons?
