# Supplementary material for: Investigating tobacco presence at retail points of sale around schools in Egypt
Source: BMC Public Health. 2025 Sep 24;25:3055. doi: 10.1186/s12889-025-24675-z (PMC12459026; doi:10.1186/s12889-025-24675-z)
Supplement: Supplementary file 2 — Supplementary Material 2. [file 12889_2025_24675_MOESM2_ESM.pdf]

1. اسم/كود الباحث
2. التاريخ
3. المحافظة
4. المدينة/ الحي
5أ. مستوى المدرسة
ب5 نوع المدرسة
6. اسم المدرسة
7. رقم المتجر
8. "ادخل العنوان (اسم الشارع ورقم المبنى)
9. نوع المتجر
(9).أ. هل سمح لك بدخول موقع المسح (المتجر)؟
(9).ب. هل بإمكانك رؤية ما بداخل المحل (المنتجات والدعاية) من خارج المحل؟
10. ما هي أنواع منتجات التبغ أو النيكوتين التي يبيعها التاجر؟
11. ما هي المنتجات المعروضة في المتجر؟
12. هل التحذير الصحي والتحذير الصحي المصوّر على علب السجائر ظاهراً؟
13. هل التحذير الصحي والتحذير الصحي المصوّر على علب تبغ الشيشة(المعسّل) ظاهراً؟
14. هل التحذير الصحي المصوّر على علب السائل الالكتروني ظاهراً؟
) ظاهراً؟15IQOS. هل التحذير الصحي المصوّر على علب سجائر التبغ المسخن (ايكوس
)، 16IQOS. هل هناك عرض لأيّ من المنتجات التالية (سجائر، سجائر الكترونية، سائل الكتروني، جهاز تبغ مسخن (ايكوس<br>)، تبغ الشيشة (معسّل)، الشيشة نفسها) في أي منطقة من المتجر؟IQOSسجائر تبغ مسخن (ايكوس
)، 17IQOS. هل هناك عرض لأيّ من المنتجات التالية (سجائر، سجائر الكترونية، سائل الكتروني، جهاز تبغ مسخن (ايكوس<br>)، تبغ الشيشة (معسّل)، الشيشة نفسها) في منطقة الكاشير في المتجر؟IQOSسجائر تبغ مسخن (ايكوس
)، سجائر تبغ مسخن 18IQOS. أيّ من المنتجات التالية (سجائر، سجائر الكترونية، سائل الكتروني، جهاز تبغ مسخن (ايكوس<br>)، تبغ الشيشة (معسّل)، الشيشة نفسها) في متناول المستهلك دون مساعدة الكاشير أو البائع في المتجر؟IQOS(ايكوس
)، سجائر تبغ مسخن 19IQOS. أيّ من المنتجات التالية (سجائر، سجائر الكترونية، سائل الكتروني، جهاز تبغ مسخن (ايكوس<br>)، تبغ الشيشة (معسّل)، الشيشة نفسها) التي في متناول المستهلك دون مساعدة وضعت على ارتفاع 1 متر عن IQOS(ايكوس<br>الأرضية؟
20. هل يعرض أكثر من علب سجائر واحدة عن كل علامة تجارية؟
21. هل لاحظت استعمال الإضاءة لإبراز أيّ من المنتجات التالية (سجائر، سجائر الكترونية، سائل الكتروني، جهاز تبغ مسخن<br>)، تبغ الشيشة (معسّل)، الشيشة نفسها)؟IQOS)، سجائر تبغ مسخن (ايكوس IQOS(ايكوس
)، 22IQOS. هل لاحظت وجود أيّ من المنتجات التالية (سجائر، سجائر الكترونية، سائل الكتروني، جهاز تبغ مسخن (ايكوس<br>)، تبغ الشيشة (معسّل)، الشيشة نفسها) على بعد 30 سم من الحلوى والمشروبات والسناك في IQOSسجائر تبغ مسخن (ايكوس<br>المتجر ؟
23. هل لاحظت أيّ من وسائل الترويج والإعلان التالية داخل المتجرالمنتجات التالية (سجائر، سجائر الكترونية، سائل الكتروني،<br>)، تبغ الشيشة (معسّل)، الشيشة نفسها) ؟ (اختر كل ما ينطبق)IQOS)، سجائر تبغ مسخن (ايكوس IQOSجهاز تبغ مسخن (ايكوس
لافتات مطبوعة (ملصقات، لافتات، منشورات، إلخ)، وليست جزءا من عرض المنتجات
لافتات مطبوعة كجزء من عرض المنتجات (خلفية، فواصل أرفف)
لافتة رقمية (شاشة، فيديو)
إعلانات مزوّدة بإضاءة
لافتة ثلاثية الابعاد
لا شيء مما ذكر
لا أعلم

24. هل لاحظت أيّ إعلانات لأيّ من المنتجات التالية (سجائر، سجائر الكترونية، سائل الكتروني، جهاز تبغ مسخن (ايكوس)، تبغ الشيشة (معسّل)، الشيشة نفسها) على ارتفاع 1 متر من الأرضية ؟IQOS)، سجائر تبغ مسخن (ايكوس IQOS
25. هل لاحظت أيّ إعلانات لأيّ من المنتجات التالية (سجائر، سجائر الكترونية، سائل الكتروني، جهاز تبغ مسخن (ايكوس)، تبغ الشيشة (معسّل)، الشيشة نفسها) تحتوي على شخصيّات كرتونية؟IQOS)، سجائر تبغ مسخن (ايكوس IQOS
26. هل لاحظت أيّ إعلانات لأيّ من المنتجات التالية (سجائر، سجائر الكترونية، سائل الكتروني، جهاز تبغ مسخن (ايكوس)، تبغ الشيشة (معسّل)، الشيشة نفسها) تحتوي على أيّ من الرسائل التالية؟ (اختر كل IQOS)، سجائر تبغ مسخن (ايكوس IQOS ما ينطبق)
يساعد في(أو يستعمل ل)الاقلاع عن التدخين
أقل ضررا من السجائر العادية
لا شيء مما ذكر
لا أعلم
27. هل لاحظت أيّ ترويج لأيّ من المنتجات التالية (سجائر، سجائر الكترونية، سائل الكتروني، جهاز تبغ مسخن (ايكوس)، تبغ الشيشة (معسّل)، الشيشة نفسها) بأيّ من الطرق التالية؟ (اختر كل ما ينطبق)IQOS)، سجائر تبغ مسخن (ايكوس IQOS
خصم على السعر (كوبونات، عرض لمدة محدودة، خصم عند شراء أكثر من عبوة)
عيّنات مجانية (مع أو بدون شراء أي بضاعة أخرى)
استبدال علب السجائر والولاعات
هدايا غير منتجات التبغ والنيكوتين الموجودة (مجانا أو حين الشراء)
حدث أو نشاط أو احتفالية برعاية علامة تجارية لمنتج تبغ أو نيكوتين
منافسات ومسابقات
مكافآت
حضور ممثل للعلامة التجارية
توجيه لموقع التواصل الاجتماعي
للموقع الالكترونيQRرمز
لا شيء مما ذكر
لا أعلم
ترويج وإعلان المنتجات
28. هل لاحظت وجود لأيّ علامة تجاريّة لأيّ من المنتجات التالية (سجائر، سجائر الكترونية، سائل الكتروني، جهاز تبغ مسخن)، تبغ الشيشة (معسّل)، الشيشة نفسها) على منتجات غير تبغيّة/ غير نيكوتين IQOS)، سجائر تبغ مسخن (ايكوس IQOS(ايكوس في المتجر (مثال: ساعات, ولّاعات سجائر, تيشرتات, كابات)؟
النكهات
29. هل يبيع المتجر أيّ من السجائر ذات النكهة (نعناع, فراولة,....إلخ)؟
30. السائل الالكتروني ذات النكهة (نعناع, فراولة, ...إلخ)؟
) ذات النكهة (نعناع, فراولة, ...إلخ)؟IQOS31. سجائر التبغ المسخن (ايكوس
لافتات أخرى
32. هل لاحظت وجود لافتة تشير إلى أنّه يمنع البيع لغير البالغين؟ اختر كل ما ينطبق
بيع السجائر الفرط
33. هل متاح بيع سجائر فرط؟
34. هل متاح بيع تبغ الشيشة (معسل) غير معبأ؟
خارج المتجر: إعلانات المنتجات
35. هل لاحظت وجود أيّ إعلانات لأيّ من المنتجات التالية (سجائر، سجائر الكترونية، سائل الكتروني، جهاز تبغ مسخن (ايكوس)، تبغ الشيشة (معسّل)، الشيشة نفسها) في الواجهة الأمامية للمتجر؟IQOS)، سجائر تبغ مسخن (ايكوس IQOS
خارج المتجر: إعلانات المنتجات
)، سجائر IQOS36. كيف يعلن عن أيّ من المنتجات التالية (سجائر، سجائر الكترونية، سائل الكتروني، جهاز تبغ مسخن (ايكوس)، تبغ الشيشة (معسّل)، الشيشة نفسها) (كما تراها من الخارج)؟ اختر كل ما ينطبقIQOSتبغ مسخن (ايكوس

لافتات مطبوعة (ملصقات، لافتات، منشورات، إلخ)، وليست جزءا من عرض المنتجات
اعلانات مع إضاءة
لافتات ثلاثية الابعاد
خارج المتجر: إعلانات المنتجات
)، سجائر تبغ مسخن 37IQOS. أيّ من المنتجات التالية (سجائر، سجائر الكترونية، سائل الكتروني، جهاز تبغ مسخن (ايكوس)، تبغ الشيشة (معسّل)، الشيشة نفسها) أو ما يتعلّق بها من منتجات يمكن ملاحظتها في داخل المتجر من خارج IQOS(ايكوس المتجر (من خلال نافذة, باب, واجهة زجاج)؟ اختر كل ما ينطبق
Power wallعرض المنتجات بما فيها ال
الإعلانات
لا شيء مما ذكر
لا ينطبق
خارج المتجر: إعلانات المنتجات
38. هل لاحظت وجود أي مقهى أو مطعم بالقرب من المدرسة يقدّم الشيشة لزبائنه؟
39. هل لاحظت وجود أي إعلانات في الواجهة الأمامية للمقهى أو المطعم لأي من المنتجات التالية؟
استكمال وإنهاء المسح في الموقع
40. في حال تمكنت من أخذ صورة دون لفت الانتباه يرجى ادراجهافي ملف الاكسيل
41. هل لديك أيّة ملاحظات إضافية عن مسح هذا الموقع (الزيارة)؟
42. هل تنصح بتصوير موقع المسح (المتجر) من قبل مصوّر محترف؟
43. نتيجة الزيارة؟
44. يرجى اختيار السبب في حال عدم اكتمال الزيارة
45. يرجى ذكر السبب
